# Diversity of mycorrhizal *Tulasnella* associated with epiphytic and rupicolous orchids from the Brazilian Atlantic Forest, including four new species

**DOI:** 10.1038/s41598-020-63885-w

**Published:** 2020-04-27

**Authors:** Emiliane Fernanda Silva Freitas, Meiriele da Silva, Everaldo da Silva Cruz, Erica Mangaravite, Melissa Faust Bocayuva, Tomás Gomes Reis Veloso, Marc-André Selosse, Maria Catarina Megumi Kasuya

**Affiliations:** 1 Departamento de Microbiologia, Universidade Federal de Viçosa, 36570–900 Viçosa, Minas Gerais State Brazil; 2 Centro Universitário Unifaminas, 36888-233 Muriaé, Minas Gerais State Brazil; 3 Institut de Systématique, Evolution, Biodiversité (ISYEB), Muséum national d’Histoire naturelle, CNRS, Sorbonne Université, EPHE, CP 39, 57 rue Cuvier, F-750055 Paris, France; 4 University of Gdańsk, Faculty of Biology, ul. Wita Stwosza 59, 80-308 Gdańsk, Poland

**Keywords:** Classification and taxonomy, Fungal biology

## Abstract

The genus *Tulasnella* often forms mycorrhizas with orchids and has worldwide distribution. Species of this genus are associated with a wide range of orchids, including endangered hosts. Initially, species identification relied mostly on morphological features and few cultures were preserved for later phylogenetic comparisons. In this study, a total of 50 *Tulasnella* isolates were collected from their natural sites in Minas Gerais, Brazil, cultured, and subjected to a phylogenetic analysis based on alignments of sequences of the internal transcribed spacer (ITS) of the nuclear ribosomal DNA. Our results, based on phylogeny, integrated with nucleotide divergence and morphology, revealed the diversity of isolated *Tulasnella* species, which included four new species, namely, *Tulasnella brigadeiroensis*, *Tulasnella hadrolaeliae*, *Tulasnella orchidis* and *Tulasnella zygopetali*. The conservation of these species is important due to their association with endangered orchid hosts and endemic features in the Brazilian Atlantic Forest.

## Introduction

Orchidaceae (or orchids) is the largest family of flowering plants, with approximately 27,000 species described^1^. The Neotropics is the region of greatest orchid diversity^2^ and approximately 205 genera and 2,650 species occur in Brazil, of which about 1,800 are endemic^3^. Many orchid species are endangered, mainly due to anthropogenic pressure and dependency between orchids and other organisms, i.e. pollinators or mycorrhizal fungi^4,5^.

Several endangered orchid species are listed in the Livro Vermelho da Flora do Brasil^6^. Among them, *Hadrolaelia jongheana* is an epiphytic orchid found in the Zona da Mata and Quadrilátero Ferrífero, two areas severely affected by anthropogenic activity. *Zygopetalum maxillare* is an epiphytic species which, although not officially endangered, grows almost exclusively in tree ferns^7^, which limits its distribution. *Cattleya cinnabarina* and *Cattleya caulescens* are rupicolous (i.e. grow on bare rocks) and endemic to the Southeastern Brazil^8^. These species belong to Brazilian Atlantic Forest, a highly diverse but endangered hotspot of biodiversity^9^. Like all orchids, they need mycorrhizal fungi for germination due to the limited reserves in seeds^10^. The symbiotic fungus supplies the embryo with carbon and other nutrients, which enable the germination and establishment of the orchid^11^. Orchids associate mainly with Basidiomycota often called rhizoctonia, a polyphyletic that includes taxa belonging to the families Sebacinaceae, Serendipitaceae, Ceratobasidiaceae and Tulasnellaceae^12,13^.

The specificity of orchid–mycorrhizal fungi varies among species^12,14^ and the distribution of mycorrhizal fungi can affect the patterns of distribution of orchids^15^. Species with low specificity for their fungal partner may be more successful in conservation strategies, such as assisted migration^8^. Despite this, specialist orchids might be widely distributed if their fungal partners are broadly distributed^14,16^. Indeed, the ecology of *Tulasnella* species orchid roots apart remains poorly known and even though they are often considered saprotrophic^11^ they may also colonize the roots of non-orchid plants^17^. The availability of compatible symbionts may directly impact the conservation of species^4^.

The genus *Tulasnella* is often observed as orchid mycorrhizal fungi in temperate and tropical regions^12,18,19^, and several isolates have been reported to increase seed germination and seedling growth^20–25^. Identification of mycorrhizal fungi in South American orchids, mostly conducted in Brazil, has often revealed *Tulasnella* symbionts: *Tulasnella* species were isolated from *Epidendrum secundum*^26,27^, *Epidendrum dendrobioides* and *Sophronits milleri*^28^, *Oeceoclades maculata*, *Epidendrum rigidum* and *Polystachya concreta*^29^, *E. rigidum* and *P. concreta*^30^. Yet little is known about *Tulasnella* in the hotspot of biodiversity of the Brazilian Atlantic Forest.

*Tulasnella* species have complex morphological characteristics, but rarely form fruitbodies *in situ* or sexual structures *in vitro*^29–33^. As morphological characteristics are not sufficient to describe *Tulasnella* species^34^, molecular approaches have been used too^32,33,35–38^. Species identification is mostly based on phylogenetic concordance of multiple unrelated genes/regions, but for this complex genus, the internal transcribed spacer (ITS) of the nuclear ribosomal DNA was shown to be highly suitable for species delimitation in *Tulasnella*^31,38^.

In a survey of cultivable mycorrhizal fungi associated with the roots of the rare-to-endangered Brazilian orchids *H. jongheana*, *C. cinnabarina*, *C. caulescens* and *Z. maxillare*, we obtained 50 isolates of *Tulasnella*. Herein, based on morphological and molecular analyses, we have evaluated the diversity of *Tulasnella* associated with these four orchids and describe potentially new *Tulasnella* species.

## Results

### *Tulasnella isolates* from Brazilian Atlantic Forest

Fifty isolates of the genus *Tulasnella* were obtained in this study (Table 1), namely, twenty isolates from *C. cinnabarina* roots, fourteen from *C. caulescens* roots, nine from *H. jongheana* (eight from Parque Estadual da Serra do Brigadeiro (PESB) and one from Parque Estadual da Serra Negra (PESN)) and seven isolates from *Z. maxillare*. As they were isolated from pelotons dissected from roots, they all are likely orchid mycorrhizal fungi. All isolates from *C. cinnabarina* and *C. caulescens* were identified as *Tulasnella calospora*, whereas isolates obtained from *H. jongheana* and *Z*. *maxillare* are described below as four new *Tulasnella* species.

**Table 1:** *Tulasnella* isolates obtained in this study. Ex-type strains are indicated in bold face.

Identity	Culture accession no.	Orchid Host	Origin	Habitat	GenBank accession no.
*Tulasnella calospora*	COAD 2850	*Cattleya caulescens*	Mariana - MG	Rupicolous	MK192009
	COAD 2851	*Cattleya caulescens*	Mariana - MG	Rupicolous	MK192010
	COAD 2852	*Cattleya caulescens*	Mariana - MG	Rupicolous	MK191991
	COAD 2853	*Cattleya caulescens*	Mariana - MG	Rupicolous	MK191993
	COAD 2854	*Cattleya caulescens*	Mariana - MG	Rupicolous	MK191994
	COAD 2855	*Cattleya caulescens*	Mariana - MG	Rupicolous	MK192007
	COAD 2856	*Cattleya caulescens*	Mariana - MG	Rupicolous	MK191995
	COAD 2857	*Cattleya caulescens*	Mariana - MG	Rupicolous	MK191996
	COAD 2858	*Cattleya caulescens*	Mariana - MG	Rupicolous	MK191997
	COAD 2859	*Cattleya caulescens*	Mariana - MG	Rupicolous	MK191998
	COAD 2860	*Cattleya caulescens*	Mariana - MG	Rupicolous	MK191999
	COAD 2861	*Cattleya caulescens*	Mariana - MG	Rupicolous	MK192000
	COAD 2862	*Cattleya caulescens*	Mariana - MG	Rupicolous	MK192005
	COAD 2863	*Cattleya caulescens*	Mariana - MG	Rupicolous	MK192003
	COAD 2864	*Cattleya cinnabarina*	Mariana - MG	Rupicolous	MK191974
	COAD 2865	*Cattleya cinnabarina*	Mariana - MG	Rupicolous	MK191975
	COAD 2866	*Cattleya cinnabarina*	Mariana - MG	Rupicolous	MK192006
	COAD 2867	*Cattleya cinnabarina*	Mariana - MG	Rupicolous	MK191976
	COAD 2868	*Cattleya cinnabarina*	Mariana - MG	Rupicolous	MK191977
	COAD 2869	*Cattleya cinnabarina*	Mariana - MG	Rupicolous	MK191978
	COAD 2870	*Cattleya cinnabarina*	Mariana - MG	Rupicolous	MK191979
	COAD 2871	*Cattleya cinnabarina*	Mariana - MG	Rupicolous	MK191980
	COAD 2873	*Cattleya cinnabarina*	Mariana - MG	Rupicolous	MK191981
	COAD 2874	*Cattleya cinnabarina*	Mariana - MG	Rupicolous	MK191982
	COAD 2875	*Cattleya cinnabarina*	Mariana - MG	Rupicolous	MK191983
	COAD 2876	*Cattleya cinnabarina*	Mariana - MG	Rupicolous	MK191984
	COAD 2877	*Cattleya cinnabarina*	Mariana - MG	Rupicolous	MK191985
	COAD 2878	*Cattleya cinnabarina*	Mariana - MG	Rupicolous	MK191986
	COAD 2879	*Cattleya cinnabarina*	Mariana - MG	Rupicolous	MK192004
	COAD 2880	*Cattleya cinnabarina*	Mariana - MG	Rupicolous	MK191987
	COAD 2881	*Cattleya cinnabarina*	Mariana - MG	Rupicolous	MK191988
	COAD 2882	*Cattleya cinnabarina*	Mariana - MG	Rupicolous	MK192008
	COAD 2883	*Cattleya cinnabarina*	Mariana - MG	Rupicolous	MK191989
*Tulasnella brigadeiroensis* sp. nov.	**COAD 2884**	*Hadrolaelia jongheana*	Araponga - MG	Epiphytic	MK192001
	COAD 3007	*Hadrolaelia jongheana*	Araponga - MG	Epiphytic	MT090025
	COAD 3008	*Hadrolaelia jongheana*	Araponga - MG	Epiphytic	MT090026
*Tulasnella hadrolaeliae* sp. nov.	COAD 2887	*Hadrolaelia jongheana*	Araponga - MG	Epiphytic	MN385724
	COAD 2888	*Hadrolaelia jongheana*	Araponga - MG	Epiphytic	MN385725
	**COAD 2889**	*Hadrolaelia jongheana*	Araponga - MG	Epiphytic	MN385726
	COAD 2890	*Hadrolaelia jongheana*	Araponga - MG	Epiphytic	MN385727
	COAD 2891	*Hadrolaelia jongheana*	Araponga - MG	Epiphytic	MN385728
*Tulasnella orchidis* sp. nov.	**COAD 2893**	*Zygopetalum maxillare*	Araponga - MG	Epiphytic	MN385729
	COAD 2894	*Zygopetalum maxillare*	Araponga - MG	Epiphytic	MN385731
	COAD 2895	*Zygopetalum maxillare*	Araponga - MG	Epiphytic	MN385730
*Tulasnella zygopetali* sp. nov.	**COAD 2896**	*Zygopetalum maxillare*	Araponga - MG	Epiphytic	MN385732
	COAD 2897	*Zygopetalum maxillare*	Araponga - MG	Epiphytic	MN385733
	COAD 2898	*Zygopetalum maxillare*	Araponga - MG	Epiphytic	MN385734
	COAD 2899	*Zygopetalum maxillare*	Araponga - MG	Epiphytic	MN385735
*Tulasnella* sp.	COAD 2885	*Hadrolaelia jongheana*	Itamarandiba - MG	Epiphytic	MK192002

### Phylogeny

The ITS alignment consisted of 93 strains (including the outgroup sequence), of which 43 are from NCBI or UNITE and 50 from this study (Tables 1 and 2) and had a total length of 583 characters (including alignment gaps). Among these, 371 characters were parsimony-informative, 419 were variable and 147 were conserved.

**Table 2:** GenBank and UNITE accession numbers of additional *Tulasnella* isolates included in the phylogenetic analysis. Ex-type strains are indicated in bold face.

Species	Strain No.	Origin	GenBank accession No.	UNITE accession No.
***Epulorhiza amonilioides***	3S	Brazil	JF907600	
*Epulorhiza amonilioides*	aero8	Brazil	KC928335	
*Epulorhiza anaticula*	UAMH 5428	Canada	EU218891	
*Epulorhiza anaticula*	13O004	South Korea	KT164598	SH1174351.08FU
*Tulasnella albida*	KC110	Unknown	AY373294	
*Tulasnella asymmetrica*	MAFF 305808 clone C001	Australia	KC152356	
*Tulasnella asymmetrica*	AL.LM4.4.1	Australia	MH134544	SH1541682.08FU
*Tulasnella bifrons*	BPI 724849	Canada	AY373290	
*Tulasnella calospora*	MAFF P305801	Ecuador	DQ388041	
*Tulasnella calospora*	MAFF P305802	Ecuador	DQ388042	
*Tulasnella calospora*	MAFF P305803	Ecuador	DQ388043	
*Tulasnella calospora*	MAFF P305804	Ecuador	DQ388044	
*Tulasnella calospora*	MAFF P305805	Ecuador	DQ388045	
*Tulasnella calospora*	FCb4	China	KC796458	SH1554832.08FU
*Tulasnella danica*	KC388	USA	AY373297	
*Tulasnella eichleriana*	KC852	Unknown	AY373292	
*Tulasnella eichleriana*	K(M)143600	United Kingdom	KC152381	
***Tulasnella irregularis***	JHW 0632	Australia	EU218889	
*Tulasnella irregularis*	D1-KT-TC-1	Thailand	GU166413	
*Tulasnella irregularis*	C3-DT-TC-2	Thailand	GU166423	SH1561236.08FU
***Tulasnella prima***	CLM159	Australia	KF476556	
*Tulasnella prima*	07033-45	Australia	HM196800	
*Tulasnella pruinosa*	DAOM 17641	Unknown	AY373295	
*Tulasnella pruinosa*	AFTOL ID610	Unknown	DQ457642	SH1549691.08FU
***Tulasnella secunda***	CLM009	Australia	KF476575	
*Tulasnella secunda*	CLM222	Australia	KF476568	
*Tulasnella* sp.	141	USA	AY373264	
*Tulasnella* sp.	10 MM-2016	USA	KU664580	
***Tulasnella sphagneti***	CLM541	Australia	KY095117	
*Tulasnella sphagneti*	CLM583	Australia	KY445922	
*Tulasnella tomaculum*	KC429	Unknown	AY373296	
***Tulasnella tubericola***	EP-15	Spain	KX929166	
*Tulasnella tubericola*	EP-1	Spain	KX774345	
*Tulasnella violea*	FO24380a	Germany	KC152439	SH1555437.08FU
*Tulasnella violea*	DC292	Germany	KC152432	
***Tulasnella warcupii***	CLM027	Australia	KF476596	
*Tulasnella warcupii*	CLM007	Australia	KF476600	
Uncultured *Tulasnella*	Clone 33tu-12	China	HM230652	
*Botryobasidium botryosum*	AFTOL ID604	Germany	DQ267124	

**Figure 1:**
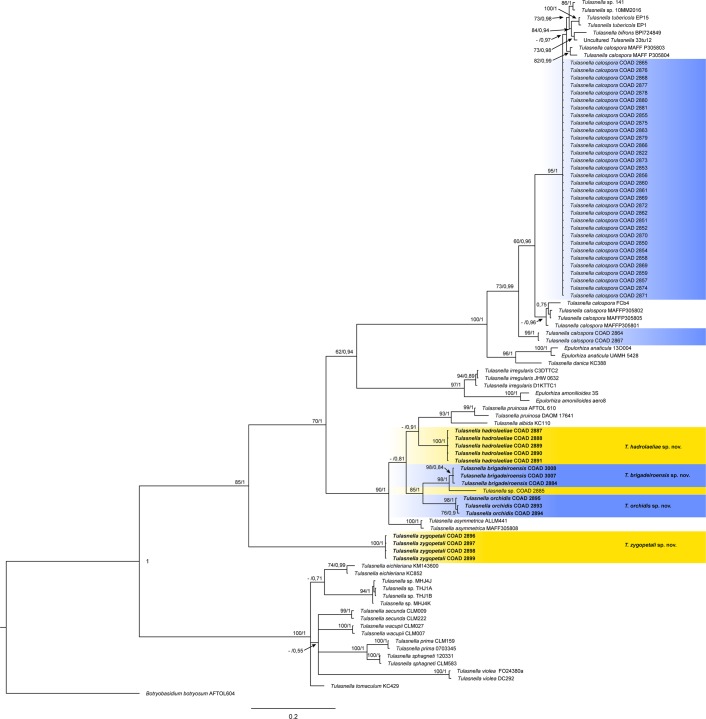
Bayesian phylogenetic tree for *Tulasnella* based on ITS alignment. Maximum likelihood bootstrap support (ML > 60) and Bayesian posterior probabilities (PP) values are indicated next to the nodes (ML/PP). Species from Brazil are in the colored block and the new species described in this paper are indicated in bold face. *Botryobasidium botryosum* (AFTOL604) was used as the outgroup.

Our phylogenetic analyses confirmed that mycorrhizal fungi isolated from the studied orchid species were *Tulasnella* (Fig. 1). Among these, four species are new in this genus and are described below, namely, *Tulasnella hadrolaeliae*, *Tulasnella brigadeiroensis*, *Tulasnella orchidis* and *Tulasnella zygopetali*. The newly proposed species are based on phylogenetic analyses, pairwise sequence divergence and morphological features (see below). The clades containing the Brazilian *Tulasnella* isolates are highlighted in the phylogenetic tree (Fig. 1).

Phylogenetically, all isolates of *Tulasnella* from *C. caulescens* and *C. cinnabarina* are grouped in a clade including *T. calospora* isolates, close to another group composed of *T. tubericola* and *T. bifrons* (Fig. 1). The new species *Tulasnella hadrolaeliae* formed a well-supported clade (Maximum likelihood (ML)/Posterior probabilities (PP) = 100/1), which is a sister group of *T. albida* and *T. pruinosa*. *Tulasnella brigadeiroensis* isolates were grouped in a monophyletic clade. *Tulasnella orchidis*, isolated from *Z. maxillare*, clustered in a sister clade to *T. brigadeiroensis* and *Tulasnella* sp. COAD 2885. Finally, isolates of *Tulasnella zygopetali* obtained from *Z. maxillare* formed a strongly supported clade (ML/PP = 100/1), distinct from other *Tulasnella* species. Although the phylogenetic analyzes indicate that *Tulasnella* sp. COAD 2885 may represent a new species, it will not be formally described here since only one isolate was obtained during our study.

### Divergence within and between clades

The Kimura-2-parameter distances between *Tulasnella* species ranged from 1.9 to 65.2% (Table 3). The divergence within *Tulasnella* species described here was lower than 0.6%. The nucleotide divergence between *Tulasnella* sp. COAD 2885 and *T. brigadeiroensis* was 7.5%, far above the 3% threshold suggested by Linde *et al*.^31^ in *Tulasnella*, and supposedly belong to two different species. For some species it was not possible to calculate the divergence within the clade, because only one isolate was used in analysis.

**Table 3:** Estimates of percentage nucleotide divergence by the Kimura-2P distances for *Tulasnella* within and between species. There was a total of 272 positions in the final dataset. All positions containing gaps and missing data were eliminated.

	Within taxa	1	2	3	4	5	6	7	8	9	10	11	12	13	14	15	16	17	18	19	20	21
1	2.2																					
2	—	8.2																				
3	1.2	16.8	14.4																			
4	0.4	16.4	14.9	2.5																		
5	—	18.1	15.1	4.3	4.4																	
6	0.4	32.9	32.7	33.7	33.6	33.5																
7	0.0	33.4	33.2	38.8	38.4	37.4	10.9															
8	—	32.9	33.9	37.3	38.2	38.7	12.6	9.0														
9	0.2	33.0	30.8	33.2	34.4	34.4	5.9	11.0	12.1													
10	—	38.2	36.6	38.8	40.4	40.1	11.7	15.9	15.9	7.5												
11	0.0	31.5	30.2	32.8	32.6	32.2	8.8	11.2	12.0	8.8	14.6											
12	0.5	34.4	32.6	34.4	35.1	33.0	8.2	10.4	13.6	5.4	10.5	8.1										
13	0.0	33.9	33.3	32.9	32.4	35.0	27.9	28.7	31.3	26.2	28.9	22.8	26.9									
14	3.4	34.4	35.0	35.9	35.1	38.1	33.6	33.1	36.1	30.1	34.8	29.1	31.0	8.6								
15	0.0	47.1	44.0	43.5	42.6	42.7	37.0	36.6	44.6	37.3	41.6	40.0	36.2	41.0	48.0							
16	0.7	58.8	57.7	60.4	61.6	63.6	49.4	48.0	52.4	46.3	51.4	54.0	49.1	51.3	52.5	50.2						
17	0.0	57.1	55.2	59.4	60.7	60.9	48.7	46.6	50.8	45.7	48.6	54.2	47.7	51.4	56.0	51.7	7.0					
18	—	57.3	56.2	59.5	60.9	61.1	48.0	46.7	49.4	45.1	50.1	51.1	47.2	50.7	53.7	48.8	3.8	5.0				
19	0.4	60.9	59.8	63.0	64.0	65.2	49.9	51.4	49.6	47.4	50.4	52.4	49.0	53.7	61.8	54.7	8.6	9.7	8.0			
20	0.0	61.3	60.2	62.6	64.1	64.3	54.6	52.4	55.3	50.6	56.1	55.7	54.3	54.4	56.7	51.0	8.8	10.7	8.2	11.8		
21	0.0	59.2	57.7	61.6	63.1	63.3	52.3	50.1	53.0	48.4	53.7	53.3	52.0	55.1	55.8	48.7	9.2	11.2	8.6	12.2	1.9	
22	0.4	62.9	62.6	59.4	60.4	59.8	53.9	49.3	51.3	52.1	54.5	51.4	52.2	52.5	56.5	54.8	18.0	17.6	16.1	19.0	21.1	20.5

1 = *Tulasnella anaticula*, 2 = *T. danica*, 3 = *T. calospora*, 4 = *T. tubericola*, 5 = *T. bifrons*, 6 = *T. asymmetrica*, 7 = *T. pruinosa*, 8 = *T. albida*, 9 = *T. brigadeiroensis*, 10 = *Tulasnella* sp. COAD 2885, 11 = *T. hadrolaeliae*, 12 = *T. orchidis*, 13 = *T. irregulares*, 14 = *T. amonilioides*, 15 = *T. zygopetali*, 16 = *T. eichleriana*, 17 = *T. secunda*, 18 = *T. tomaculum*, 19 = *T. wacupii*, 20 = *T. prima*, 21 = *T. sphagneti*, 22 = *T. violea*.

### Taxonomy

**Figure 2:**
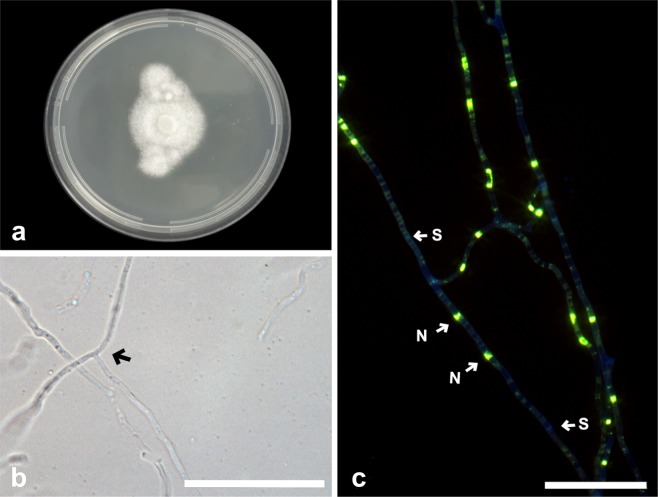
*Tulasnella brigadeiroensis* (COAD2884). (**a**) Eight-day-old PDA culture. (**b**) Hyphae with branching at right angles. (**c**) Hyphae stained with SYBR Green I showing binucleate cells (N = nuclei; S = septa). Bars = 50 µm.

***Tulasnella brigadeiroensis*** E.F.S. Freitas, Meir. Silva & M.C.M. Kasuya, sp. nov. (Fig. 2)

*Mycobank*: MB832785

*Etymology*:— Referring to Parque Estadual Serra do Brigadeiro, where the type species was isolated.

*Diagnosis: Tulasnella brigadeiroensis* is phylogenetically closely related to *T. orchidis*. In a comparison of the 583 ITS nucleotides, *T. brigadeiroensis* differs from *T. orchidis* by 47 bp (8.1%).

*Type:*—BRAZIL: Minas Gerais: Parque Estadual Serra do Brigadeiro, isolated from roots of the orchid *Hadrolaelia jongheana*, February 2018, E.F.S. Freitas (holotype VIC47299, ex-type culture COAD2884).

*Description:* Colonies on PDA attaining 31 mm diam after 8 d at 25 °C, white to cream, with undulate and submersed edge, aerial mycelium present. Reverse of the colony white to cream. Hyphae are regularly septate with branching at right angles, 1.5–2.5 µm diam ($\bar{{\rm{X}}}$ ± SD = 2 ± 0.3 μm), hyaline, with binucleate cells. Molinioid cells not observed. Sexual morph not observed.

*Substrate or host*: Roots of *Hadrolaelia jongheana*.

*Additional material examined*.—BRAZIL: Minas Gerais: Parque Estadual Serra do Brigadeiro, from roots of *Hadrolaelia jongheana*, October 2019, E.F.S. Freitas (COAD3007, COAD3008). This species was isolated three times from two roots. There was no difference between the morphology of the isolates.

**Figure 3:**
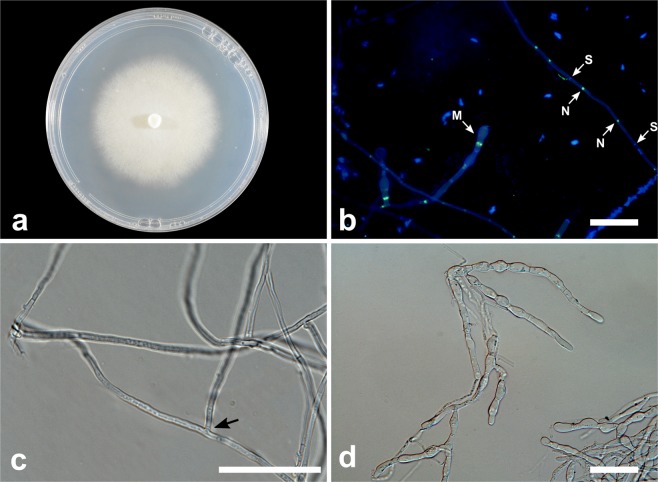
*Tulasnella calospora* (COAD2869). (**a**) Eight-day-old PDA culture. (**b**) Hyphae stained with SYBR Green I showing binucleate cells (M= monilioid cell; N = nuclei; S = septa). (**c**) Hyphae with branching at right angles. (**d**) Monilioid cell chains in CMA. Bars = 50 µm.

***Tulasnella calospora*** Juel, Bih. K. svenska Vet-Akad. Handl. 23: 23 (1897). (Fig. 3)

*Description:* Colonies on PDA attaining 45–67 mm diam after 8 d, at 25 °C, white to cream, with undulate and submersed edge, some cultures showing aerial mycelium. Hyphae from cultures are regularly septate, with branching at right angles, 3–4 µm diam ($\bar{{\rm{X}}}$ ± SD = 3.5 ± 0.3 μm), hyaline, with binucleate cells. Molinioid hyaline, barrel to elongated barrel-shaped, in branched chains with more than five cells. Sexual morph not observed.

*Substrate or host*: Roots of *Cattleya caulescens* and *Cattleya cinnabarina.*

*Additional material examined*—BRAZIL. Minas Gerais, Mariana, Mina da Alegria, Vale S.A., isolated from roots of *Cattleya caulescens*, COAD 2850–COAD2863; and from roots of *Cattleya cinnabarina*, COAD2864–2883, 2010, Bocayuva, M.F. There was no difference between the morphology of the isolates.

**Figure 4:**
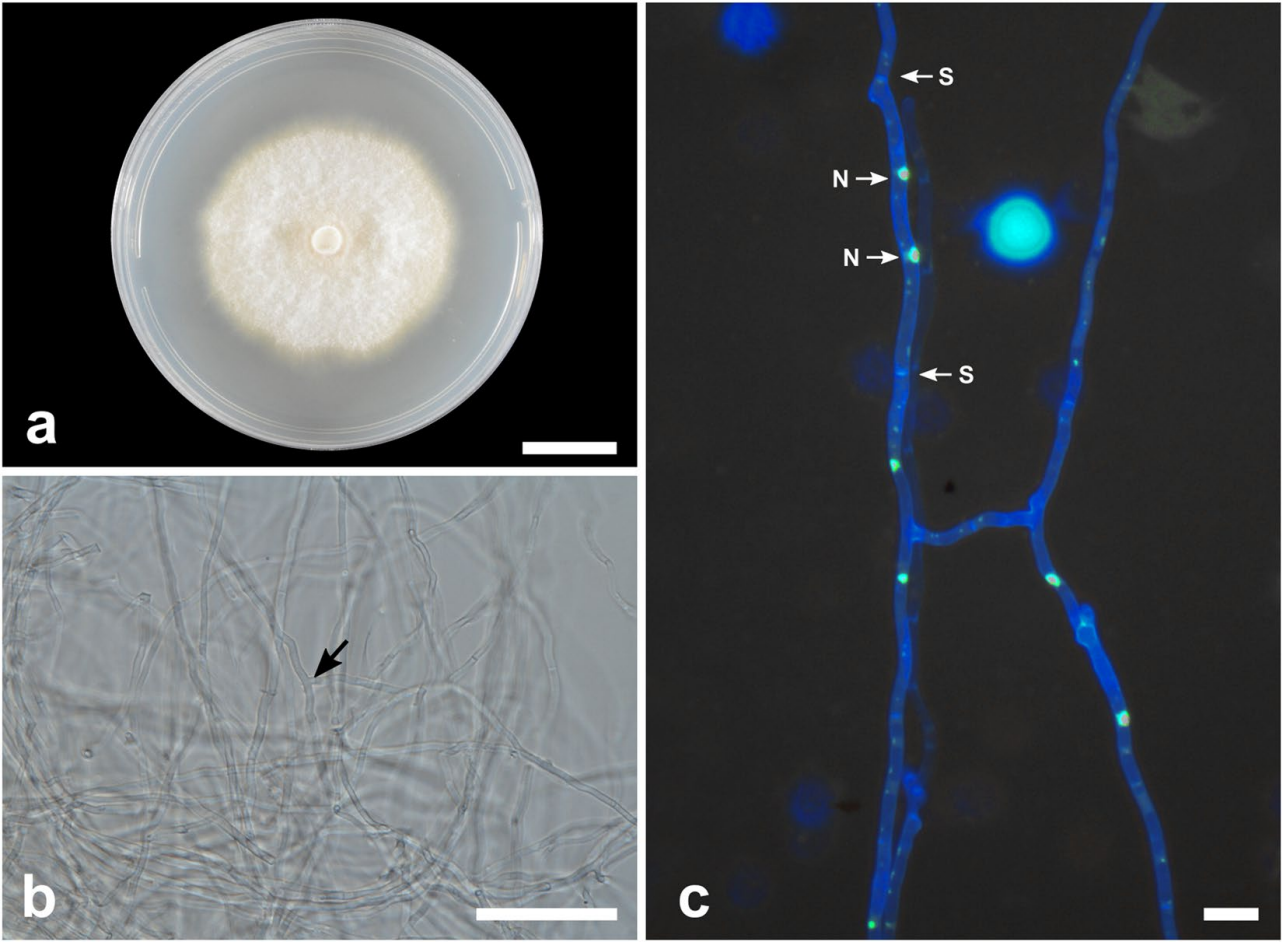
*Tulasnella hadrolaeliae* (COAD2889). (**a**) Thirty-day-old PDA culture. (**b**) Hyphae with branching at right angles. (**c**) Hyphae stained with SYBR Green I showing binucleate cells (N = nuclei; S = septa). Bars: B = 50 µm; C = 10 µm.

***Tulasnella hadrolaeliae*** E.F.S. Freitas, Meir. Silva & M.C.M. Kasuya, sp. nov. (Fig. 4)

*Mycobank*: MB832786

*Etymology*: — Name derived from the plant host genus *Hadrolaelia*.

*Diagnosis: Tulasnella hadrolaeliae* is phylogenetically closely related to *T. albida* and *T. pruinosa*. In a comparison of the ITS nucleotides, *T. hadrolaeliae* differed from *T. albida* by 64 bp (11%) and from *T. pruinosa* by 73 bp (12.5%).

*Type:*—BRAZIL: Minas Gerais: Parque Estadual Serra do Brigadeiro, isolated from roots of orchid *Hadrolaelia jongheana*, February 2018, E.F.S. Freitas (holotype VIC47304, ex-type culture COAD2889).

*Description:* Colonies on PDA showed very slow-growing (56–59 mm diam after 30 d at 25 °C), white to cream, showing concentric rings, with undulate and submersed edge, aerial mycelium present. Reverse of the colony white to cream. Hyphae are regularly septate with branching at right angles, 2–3.5 µm diam ($\bar{{\rm{X}}}$ ± SD = 2.5 ± 0.3 μm), hyaline, with binucleate cells and thin-walled. Molinioid cells not observed. Sexual morph not observed.

*Substrate or host*: Roots of *Hadrolaelia jongheana*.

*Additional material examined*.—BRAZIL: Minas Gerais: Parque Estadual Serra do Brigadeiro, from roots of *Hadrolaelia jongheana*, February 2018, E.F.S. Freitas (COAD2887, COAD2888, COAD2890, COAD2891). This species was isolated five times from three roots. There was no difference between the morphology of the isolates.

**Figure 5:**
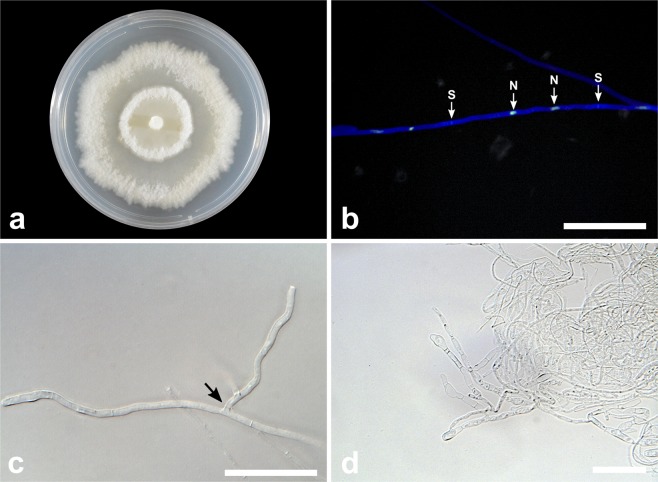
*Tulasnella orchidis* (COAD2893). (**a**) Fourteen-day-old PDA culture. (**b**) Hyphae stained with SYBR Green I showing binucleate cells (N = nuclei; S = septa). (**c**) Hyphae with branching at right angles. (**d**) Monilioid cell chains in CMA. Bars = 50 µm.

***Tulasnella orchidis*** E.S. Cruz, E.F.S. Freitas, Meir. Silva & M.C.M. Kasuya, sp. nov. (Fig. 5)

*Mycobank*: MB832787

*Etymology*:— Name derived from the nature of host, an orchid, from which it was isolated.

*Diagnosis: Tulasnella orchidis* differs from *T. brigadeiroensis* by the culture characteristics on PDA, colonies forming concentric rings with undulate edge, whereas *T. brigadeiroensis* show uniform colonies with regular edge. In a comparison of the 583 ITS nucleotides, *T. orchidis* differed from *T. brigadeiroensis* by 47 bp (8%).

*Type:*—BRAZIL: Minas Gerais: Parque Estadual Serra do Brigadeiro, isolated from roots of *Zygopetalum maxillare*, February 2019, E.S. Cruz (holotype VIC47308, ex-type culture COAD2893).

*Description:* Colonies on PDA attaining 62–71 mm diam after 14 d, at 25 °C, white to cream, with undulate and submersed edge, showing concentric rings, no formation of aerial mycelium. Reverse of the colony white to cream. Hyphae are regularly septate with branching at right angles, 2.5–4.5 µm diam ($\bar{{\rm{X}}}$ ± SD = 3.5 ± 0.5 μm), hyaline, with binucleate cells and thin-walled. Molinioid cells hyaline, barrel to elliptical-shaped, 5–11.5 µm diam ($\bar{{\rm{X}}}$ ± SD = 8 ± 2 μm) and in branched chains. Sexual morph not observed.

*Substrate or host*: Roots of *Zygopetalum maxillare*.

*Additional material examined*.—BRAZIL: Minas Gerais: Parque Estadual Serra do Brigadeiro, from roots of *Zygopetalum maxillare*, February 2019, E.S. Cruz (COAD2894, COAD289). This species was isolated three times from the same root. There was no difference between the morphology of the isolates.

**Figure 6:**
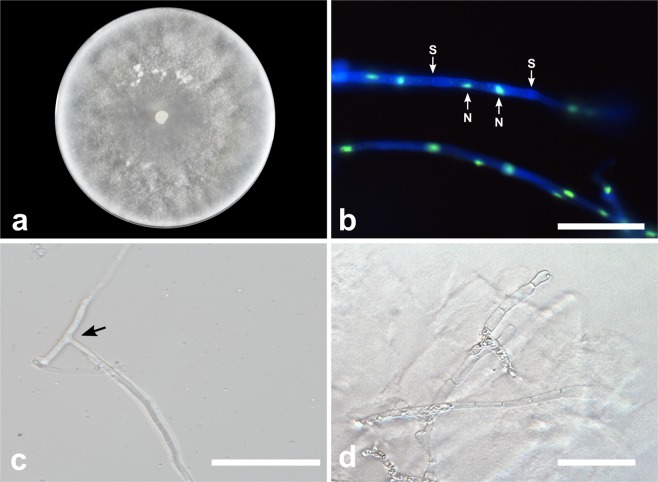
*Tulasnella zygopetali* (COAD2896). (**a**) Eight-day-old PDA culture. (**b**) Hyphae stained with SYBR Green I showing binucleate cells (N = nuclei; S = septa). (**c**) Hyphae with branching at right angles. (**d**) Monilioid cell chains in CMA. Bars = 50 µm.

***Tulasnella zygopetali*** E.S. Cruz, E.F.S. Freitas, Meir. Silva & M.C.M. Kasuya, sp. nov. (Fig. 6)

*Mycobank*: MB832789

*Etymology*: — Name derived from the plant host genus *Zygopetalum*, from which it was first collected.

*Diagnosis: Tulasnella zygopetali* is phylogenetically different from other *Tulasnella* species. Morphologically, *T. zygopetali* differs from other *Tulasnella* species described here as it has wider hyphae (3–6 µm diam) and monilioid cells (6.5–12.5 µm diam). In a comparison of the 583 ITS nucleotides, *T. zygopetali* differed from *T. brigadeiroensis* by 134 bp (23%), from *T. hadrolaeliae* by 148 bp (25.4%) and from *T. orchidis* by 135 bp (23%).

*Type:*—BRAZIL: Minas Gerais: Parque Estadual Serra do Brigadeiro, isolated from roots of *Zygopetalum maxillare*, February 2019, E.S. Cruz (holotype VIC47311, ex-type culture COAD2896).

*Description:* Colonies on PDA attaining 86 mm diam after 8 d, at 25 °C, white to cream, with regular and submersed edge, dense aerial mycelium. Reverse of the colony white to cream. Hyphae are regularly septate with branching at right angles, 3–6 µm diam ($\bar{{\rm{X}}}$ ± SD = 4 ± 0.9 μm), hyaline, with binucleate cells and thin-walled. Molinioid cells hyaline, elongated barrel-shaped, 6.5–12.5 µm diam ($\bar{{\rm{X}}}$ ± SD = 10 ± 1.5 μm), in branched chains with more than five cells. Sexual morph not observed.

*Substrate or host*: Roots of *Zygopetalum maxillare*.

*Additional material examined*—BRAZIL: Minas Gerais: Parque Estadual Serra do Brigadeiro, from roots of *Zygopetalum maxillare*, February 2019, E.S. Cruz (COAD2897, COAD2898, COAD2899). This species was isolated four times from the same root. There was no difference between the morphology of the isolates.

## Discussion

We investigated *Tulasnella* species associated with the roots of four Brazilian orchids from different vegetations of the Atlantic Forest, where this fungal genus is little known. A previous study of the same area, based only on the molecular approach, observed high fungal community diversity in roots of *H. jongheana*, *C. caulescens* and *C. cinnabarina* orchids, but no *Tulasnella* was identified^8^. The authors suggested that *Tulasnella* sequences were not detected due to the primers used. Indeed, universal fungal primers such as ITS1F/ITS4 often do not detect *Tulasnella* species due to a high rate of molecular evolution of nuclear rDNA genes in this genus^35,39^.

The genus *Tulasnella* (Tulasnellaceae) was described in 1888 by Schröter, with *Tulasnella lilacina* J. Schröt. as the type species, and nowadays there are 73 accepted species in Index Fungorum^40^. Due to the lack of molecular data from the type specimen, many *Tulasnella* species are described only by morphological-based approaches^38^. Morphological characters, such as size and shape of hyphae, basidia, sterigmata and basidiospore, when used alone, may lead to incorrect species identification^34^, e.g. because they are affected by cultural conditions. For species delimitation, we have combined both molecular and morphological data as recommended by Cruz *et al*.^34,36^, using ITS as suggested by Linde *et al*.^38^.

Among the species of the genus *Tulasnella, T. calospora* is considered as a nearly universal orchid symbiont^41^. It has been isolated from orchids in Asia^42,43^, Australia^44,45^, Europe^46^ and South America^47,48^. However, the definition of *T. calospora* species is still unclear, since phylogenies have shown taxonomic problems concerning this species^35^. In Brazil, *T. calospora* was obtained from the roots of the orchids *Oeceoclades maculata*^29^, *Epidendrum secundum*, *Acianthera limae* and *Polystachya concreta*^48^ in the Zona da Mata and Quadrilátero Ferrífero regions of the state of Minas Gerais. Herein, *T. calospora* was isolated from *C. caulescens* and *C. cinnabarina* roots also sampled in the Quadrilátero Ferrífero region. These results suggest that *T. calospora* is a nonspecific orchid symbiont broadly distributed in the studied region.

The present study also yielded information for four species, which likely are only a small fraction of the unknown *Tulasnella* species diversity*. Tulasnella hadrolaeliae* and *T. brigadeiroensis* are mycorrhizal fungi isolated from pelotons in the roots of *H. jongheana*, an endangered epiphytic orchid. *Tulasnella brigadeiroensis* was collected at two different times: first (February 2018) just one isolate was obtained, and second (October 2019) two additional isolates of the new species *T. brigadeiroensis* were collected. *Tulasnella zygopetali* and *T. orchidis* were isolated from pelotons from the same individual of *Zygopetallum maxillare*. *Zygopetalum maxillare* is an epiphytic orchid with high specificity in a host tree relationship^7^. In PESB, *Z. maxillare* grows exclusively on the stems of tree ferns.

The new *Tulasnella* species studied here were described using a polyphasic approach. Phylogenetically, *T. hadrolaeliae* formed a sister clade with *T. albida* and *T. pruinosa*. However, the definition of the phylogenetic species of *T. albida* cannot be confirmed due to the absence of molecular data from the type specimen^49^. Additionally, morphological characters cannot distinguish *T. albida* and *T. pruinosa*^34^. Therefore, as for *T. calospora*, molecular data from the type specimen are required to confirm the delimitation of the species *T. albida* and *T. pruinosa*^49^.

*Tulasnella brigadeiroensis* and *T. orchidis* formed well-supported sisters clades. *Tulasnella brigadeiroensis* and *Tulasnella* sp. COAD 2885 showed high percentage sequence divergence between clades (7.5%). This value is higher than the 3% sequence divergence cut-off value proposed for species delimitation^50^ or 3–5% divergence used for *Tulasnella* species^38^. Regarding the other new species described here, the interspecific nucleotide divergence ranged from 5.4 to 41.6%. These values are comparable to or even higher than those found in previous studies on *Tulasnella*^33,34,38^.

Knowledge of the diversity of orchid mycorrhizal fungi is important for successful conservation strategies^4^, together with their maintenance in culture collection. Our study contributes to the description of diversity of *Tulasnella* associated with orchids of the Brazilian Atlantic Forest, which is relevant for conservation of these orchids and for knowledge of fungal richness in this hotspot of biodiversity. Further studies are required to verify the potential of new species to support seed germination, seedling development and, consequently, orchid conservation programs.

## Conclusions

Phylogenetic analyses, integrated with nucleotide divergence and morphological characteristics, showed the diversity of *Tulasnella* species associated with orchids of the Brazilian Atlantic Forest, including the description of four novel *Tulasnella* species. This is the first study using a polyphasic approach to the description of *Tulasnella* in Brazil, and it suggests that further studies will uncover more diversity. The cultivation of these species may help the strategies of conservation of endangered Brazilian orchids.

## Methods

### Sample collection and isolates

**Figure 7:**
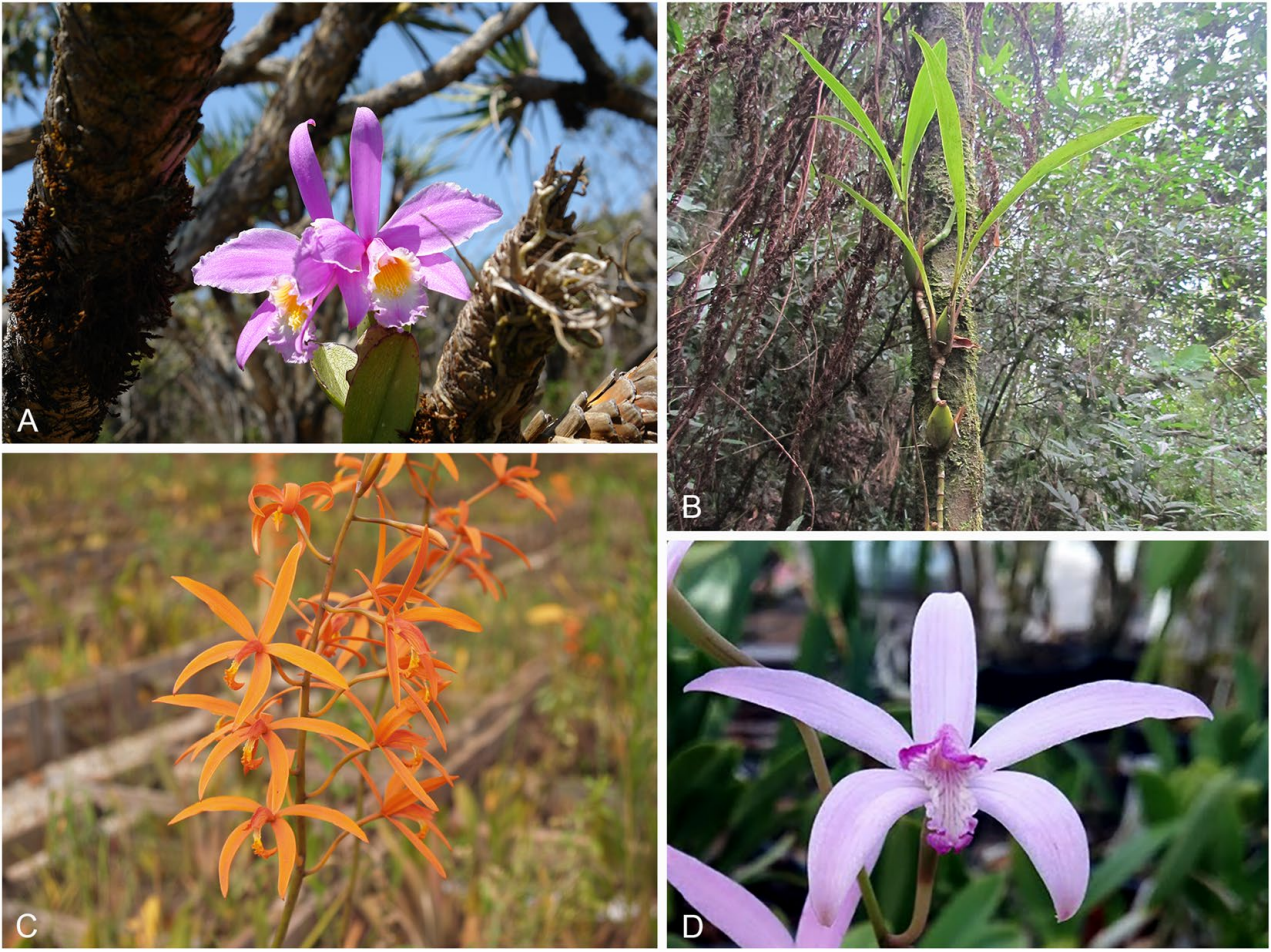
Investigated orchids: (**a**), flower of *Hadrolaelia jongheana*; (**b**), *Zygopetalum maxillare*; (**c**), flower of *Cattleya cinnabarina*; (**d**), flower of *Cattleya caulescens*.

Root samples of the epiphytic orchid *H. jongheana* were collected from the PESB (Araponga – MG, Brazil) and PESN (Itamarandiba – MG, Brazil) (Fig. 7). *Zygopetalum maxillare* samples were also obtained from PESB, while *C. cinnabarina* and *C. caulescens* were sampled from iron mining areas in the Quadrilátero Ferrífero region (Mariana – MG, Brazil) (Fig. 7). Apparently healthy roots were analyzed at the Laboratório de Associações Micorrízicas (DMB/UFV). The root samples were gently washed under running tap water, cut into pieces of transversal root fragments, 2–3 mm thick, surface-sterilized in 70% ethanol for 1 min, 2% sodium hypochlorite for 3 min, followed by two successive rinses of sterile distilled water. These fragments were then examined under a stereomicroscope, after slicing into several thin transversal slices. Cells containing pelotons were placed on potato dextrose agar (PDA) medium without antibiotics and then incubated at 25 °C in the dark. Axenic cultures were preserved on rice grains in an ultrafreezer at −72 °C or silica gel and were deposited in the Coleção Oswaldo Almeida Drummond collection (COAD) at the Universidade Federal de Viçosa. Representative specimens were deposited at the Fungarium of the Universidade Federal de Viçosa (VIC).

### Morphology

The fungus and colony characteristics were described from cultures grown on PDA at 25 °C in the dark for 1–4 weeks depending on their growth rate. Measurements of colony diameters were taken using digital calipers. Color terminology followed Rayner^51^. The nuclear condition was observed from young hyphae after staining with SYBR Green I according to Meinhardt *et al*.^52^. The isolates were transferred to Corn Meal Agar (CMA) medium and incubated at 25 °C in the dark, for 4–6 weeks, to induce monilioid cell formation^29^. Observations, measurements and photographic images of microscopic fungal structures were recorded using an Olympus BX53 light microscope, with an Olympus Q-Color5TM digital high-definition color camera and differential interference contrast (DIC) illumination. Adobe Photoshop CS5 was used for the final editing of the acquired images and photographic preparations.

### DNA extraction, PCR amplification and sequencing

The genomic DNA was extracted from fungal mycelia grown on PDA at 25 °C for 4 weeks, using the Nucleospin® Soil (MACHEREY-NAGEL GmbH & Co. KG), in accordance with the manufacturer’s instructions. The nuclear ribosomal internal transcribed spacer (ITS) region was amplified using primer pairs ITS1 and ITS4^53^. Each polymerase chain reaction (PCR) was performed in 50 µL containing 10–20 ng of DNA template, 1× Taq buffer, 2 mM MgCl_2_, 0.2 μM of each primer, 0.4 mM of each dNTP, and 1.0 U Taq DNA polymerase (Cellco Biotec do Brasil Ltda, São Paulo, Brazil). PCR was carried out using a MyCyclerTM Thermal Cycler (Bio-Rad Laboratories B.V., Veenendal, The Netherlands) with an initial denaturation at 95 °C, for 2 min, followed by 39 PCR cycles (denaturation at 95 °C for 1 min; annealing at 50 °C for 1 min; extension at 72 °C for 1 min) before a final extension at 72 °C for 10 min.

The PCR products were visualized on 1% agarose gels stained with ethidium bromide to assess product size and quality, purified and then sequenced from the two strands using the primers ITS1 and ITS4^53^. Consensus sequences were generated using the MEGA v.7.0.26 software tool^54^. All sequences were checked manually, and nucleotides with ambiguous positions were clarified using both primer direction sequences. The sequences were deposited in GenBank (see accession numbers in Table 1).

### Phylogenetic analyses

Consensus sequences were compared against NCBI’s GenBank nucleotide databases by using the BLASTn algorithm. The most similar sequences were downloaded in FASTA format and aligned with our sequences by using the MAFFT v. 7 online portals^55^. The resulting sequence alignments were manually checked and adjusted in MEGA v.7.0.26 software tool^54^.

Bayesian inference (BI) analyses employing a Markov Chain Monte Carlo method were performed on all sequences. Nucleotide substitution models were determined using the MrModeltest 2.3 program^56^ and, once the likelihood scores had been calculated, the models were selected according to the Akaike information criterion (AIC). The results of MrModeltest recommended a GTR + G model for ITS, and a dirichlet (1,1,1,1) state frequency distribution and a gamma distributed rate variation were set. The phylogenetic analysis was performed using the CIPRES web portal^57^ and the MrBayes program v.3.1.1^58^. Two sets of four MCMC chains were run simultaneously, starting from random trees for 1,000,000 generations and sampling every 1,000th generation. The first 25% of the trees were discarded as the burn-in phase for each analysis. Posterior probabilities^59^ were determined from the remaining trees and are presented on the left of each node. Maximum likelihood (ML) analysis was implemented using the RAxML-HPC v.8 on XSEDE (8.2.12) available on the CIPRES web portal. Parameters for maximum likelihood were set to rapid bootstrapping and the analysis was carried out using 1000 replicates. Alignments and trees were deposited in TreeBASE (http://treebase.org/treebase-web/) (25158). The trees were visualized in FigTree V1.4.4^60^ and the layout of the tree for publication was done using Adobe Illustrator v. CC.

### Divergence between clades and haplotype network

In order to assess the sequence divergence between and within the clades obtained in the phylogeny tree, the Kimura-2-parameter distances were calculated as implemented in MEGA v.7.0.26^61^. The analysis involved 85 nucleotide sequences. All positions containing gaps and missing data were eliminated. There was a total of 272 positions in the final dataset.

## Data Availability

All materials examined were deposited in the public culture collection of the Coleção Oswaldo Almeida Drummond (COAD), of the Universidade Federal de Viçosa. Alignments and tree files generated during the current study are available at TreeBASE (accession https://www.treebase.org/treebase-web/home.html; study 25158). All sequence files are available from the GenBank database. The complete list of accession numbers is included in Table 1. They will be made available to the public after the publication of the paper.
